# Correction: Dendritic fibrous nanosilica-supported dendritic IL/Ru(ii) as photocatalysts for the dicarbofunctionalization of styrenes with carbon dioxide and amines

**DOI:** 10.1039/d1ra90122b

**Published:** 2021-06-23

**Authors:** Can Liu, Jalal Rouhi

**Affiliations:** School of Electronic Engineering, Xi’an Shiyou University Xi’an 710065 China liucanxk@126.com; Faculty of Physics, University of Tabriz Tabriz 51566 Iran jalalrouhi@gmail.com

## Abstract

Correction for ‘Dendritic fibrous nanosilica-supported dendritic IL/Ru(ii) as photocatalysts for the dicarbofunctionalization of styrenes with carbon dioxide and amines’ by Can Liu *et al.*, *RSC Adv.*, 2021, **11**, 9933–9941, DOI: 10.1039/D0RA10729H.

The authors regret that, in the original article, the given funding information was added in error and does not belong to this article. There is no funding information that applies to this article.

The Royal Society of Chemistry apologises for these errors and any consequent inconvenience to authors and readers.

## Supplementary Material

